# Resilience and Flexibility for Clinical Nurses: A Latent Class Analysis

**DOI:** 10.1155/2024/6171305

**Published:** 2024-05-20

**Authors:** Shuang Zhao, Zeyu Zhang, Xiaocui Duan, Yujiao Shao, Fuzhi Wang, Yongxia Chen, Congyan Yang, Lingling Chen, Fei Wang, Jiaoping Zhang, Hailing Zhang, Xiumu Yang, Changjiang Yuan

**Affiliations:** ^1^School of Nursing, Bengbu Medical University, Bengbu, Anhui, China; ^2^School of Health Management, Bengbu Medical University, Bengbu, Anhui, China; ^3^The First Affiliated Hospital of Bengbu Medical University, Bengbu, Anhui, China; ^4^The Second Affiliated Hospital of Bengbu Medical University, Bengbu, Anhui, China; ^5^Bengbu Third People's Hospital of Bengbu Medical University, Bengbu, Anhui, China; ^6^Bengbu First People's Hospital, Bengbu, Anhui, China; ^7^The First Affiliated Hospital of University of Science and Technology of China, Hefei, Anhui, China; ^8^General Practice Education and Development Center, Bengbu Medical University, Bengbu, Anhui, China

## Abstract

**Aim:**

To explore potential resilience and psychological flexibility patterns in nurses and analyze the effects of related factors such as growth mindset and professional recognition of categories.

**Background:**

Resilience and psychological flexibility can help nurses resist occupational pressure and play essential roles in promoting personal growth and professional development.

**Methods:**

A latent category approach was used to examine the patterns of heterogeneity in resilience and flexibility among 805 nurses. Differences in the influences related to resilience and flexibility were analyzed using univariate and multivariate logistic regressions, with demographic information, growth mindset, and career recognition as covariates.

**Results:**

Participants were divided into three potential categories: toughness-flexible (32.8%), power-deficit-emotional (23.1%), and toughness-rigid (44.1%). The results of multivariate logistic regression analysis showed that monthly income, mode of employment, growth mindset, and professional identity were influential factors in the potential categories of nurse resilience and flexibility.

**Conclusion:**

One cohort of nurses had high resilience and low flexibility, and psychological rigidity was related to the fact that the monthly income was less than RMB 5,000 and the contractual mode of employment. An excellent growth mindset and a high professional identity indicate that nurses are resilient and flexible. *Implications for Nursing Management*. Hospitals and nursing managers should pay attention to nurses' different career development needs and implement appropriate safeguards.

## 1. Introduction

There is no single definition of resilience or mental flexibility. An extensive review of the empirical literature summarizes resilience into five themes: overcoming adversity, adaptation and adjustment, prevalence, good mental health, and positive personal responses to challenges [1]. Psychological flexibility is the management of actions in a way that facilitates the pursuit of one's goals or values as appropriate to the situation's needs [2]. In resilient shield theory [3], several indicators are interwoven to form a protective shield to help people withstand adversity. This clearly explains the theoretical system developed by many factors and flexibilities, in which the flexibility of the thinking layer allows for the transformation of cognition. Overall, resilience positively correlated with coping. Psychological flexibility, in contrast, is accompanied by goal orientation and action management. Both resilience and flexibility in terms of favorable outcomes mean beating the odds, but there may be differences in the process involved and the result of the choices made.

The nursing community is a high-pressure occupational group, and nurses' mental health in China and other countries has received particular attention [4]. Work pressure, burnout, moral dilemmas, and vicarious trauma often lead to anxiety, depression, and other negative psychological aspects of this group [5–7], thus, laying hidden dangers to the quality of nursing services and even casting doubt on the value of the nursing profession [8]. When nurses face stress and challenges over time, better resilience helps them cope and adapt to adversity. However, psychological flexibility serves as a guiding light, telling the nurse the direction or goal for which they should strive. Better resilience and flexibility with greater adaptability positively impacted nurses' psychological, physical, and occupational functioning.

Resilience is dynamically variable and multifactorial from resource-, outcome-, and process-based perspectives [9]. The process of coping with stress and challenges reflects the underlying characteristics of individuals. Although many studies have demonstrated a significant positive correlation between resilience and psychological flexibility, it is unknown whether more resilient individuals possess better psychological flexibility. Latent class analysis can categorize the study population based on probability, significantly differentiating between groups and allowing heterogeneous individuals to exhibit characteristics that differ from other categories [10]. This is an excellent approach for exploring differences in resilience and flexibility within the same group of nurses. Compared to clustering algorithms, latent class analysis reduces human-induced errors and can be used to test more complex variable relationships. Therefore, this study aimed to explore resilience and psychological flexibility patterns among clinical nurses.

**Figure 1:**
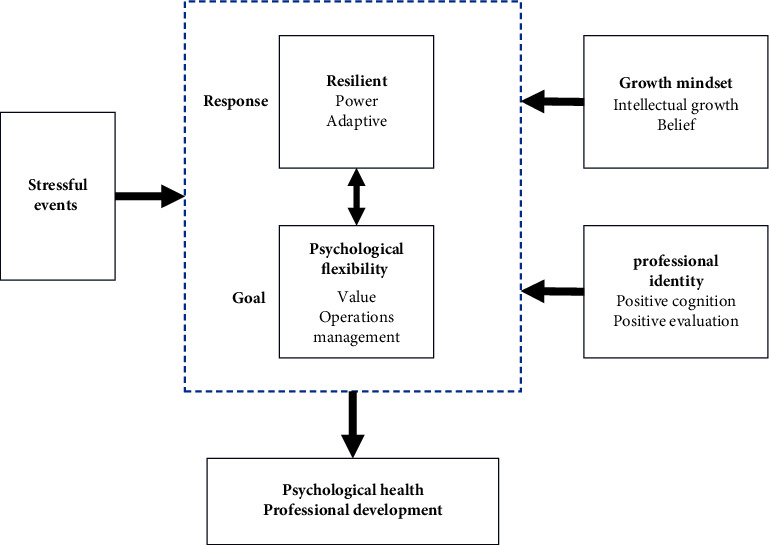
Combined framework of resilience and psychological flexibility models. In the face of stressful events, resilience and psychological flexibility comprise different psychological characteristics of a positive coping model with goal orientation. Resilience and psychological flexibility interact in the model and form a heterogeneous pattern by their different demographic characteristics. A growth mindset and professional identity had an influential role in the model. In summary, the resilience and psychological flexibility model influences mental health and professional development.

The second aim was to elucidate the factors influencing nurses' resilience and psychological flexibility. The growth mindset theory hypothesizes that belief in changing our intellect and mindset can influence how we handle challenges and define goals [11]. The motivation involved involves a complex interaction between goal orientation and mindset and has a wide range of applications in education. Michael reviewed 27 medical professional education articles on growth mindset. He noted that one of its potential benefits is that it provides people with emotional and psychological support, increased resilience, improved mental health, and self-confidence [12]. Professional identity is a positive perception and evaluation of the occupation in which you work [13]. Affirmation of the profession likewise affects the psychological state of the nurses themselves, their personal career development, quality nursing care, and the advancement of the professional discipline [14]. Nurses' resilience is significantly associated with their professional identity [15]. Higher resilience corresponds to a more positive career perception [16].  Figure 1 illustrates this study's portfolio framework.

Therefore, we propose the following hypotheses: (a) there is heterogeneity in patterns of resilience and psychological flexibility among clinical nurses, (b) a growth mindset can influence nurses' patterns of resilience and psychological flexibility, and (c) professional identity can influence nurses' patterns of resilience and psychological flexibility. Exploring models of nurse resilience, psychological flexibility, and related influences can provide hospitals and nursing administrators with new perspectives and evidence for nurses' mental health management and career development.

## 2. Method

### 2.1. Study Participants

In August 2023, nurses from five public hospitals at level II and above in Anhui Province were selected as survey respondents using convenience sampling. Inclusion criteria encompassed the following: (1) possessing a nursing license; (2) working as a nurse for more than one year; and (3) informed consent to this study; exclusion criteria included the following: leave of absence such as vacation and further training.

### 2.2. Measurement

#### 2.2.1. General Information Questionnaire

General information included age, sex, education, marital status, number of children, title, position, duration of employment, education, mode of employment, monthly income, and hospital grade status.

#### 2.2.2. Simplified Version of the Psychological Resilience Scale

The original scale was the Connor–Davidson Resilience Scale [17]. The study was tested using the Chinese version of Laura Campbell-Sills [18], simplifying the 10-item entry [19]. The scale is based on a 5-point Likert scale with a positive scoring principle. It has shown good reliability and validity for testing mental health in the Chinese population. The Cronbach's alpha coefficient for this scale in this study was 0.933.

#### 2.2.3. Acceptance and Action Questionnaire Second Edition

The second version of the Acceptance and Action Questionnaire (AAQ II) was developed by Hayes et al. and later adapted by Bond et al. [20]. The study used the Chinese version of Cao et al. [21], with 7 entries. Currently, there are more satisfactory applications for different Chinese populations. The scale measures acceptance and action on a 7-point Likert scale (from 1 = “never” to 7 = “always”), with higher total scores resulting in lower levels of psychological flexibility. The Cronbach's alpha coefficient for this scale in the present study was 0.973.

#### 2.2.4. Growth Mindset Scale

This study uses the Growth Mindset Scale revised by Zhu et al. [22] in the context of Chinese culture, developed by Dweck et al., comprising three items. The scale is based on a 6-point Likert scale, with scores of 1–6 indicating complete agreement with complete disagreement; the higher the score, the better the level of growth mindset. The Cronbach's *α* for this study was 0.957.

#### 2.2.5. Nurses' Professional Identity Rating Scale

The original scale was obtained from the University of Tokyo, Japan, and a version translated by Fangli Deng from China was used in this study [23]. The scale was merged into five dimensions based on Chinese cultural conventions: sense of self-efficacy and grasp, sense of congruence, sense of self-determination, sense of patient and organizational influence, and sense of meaningfulness. The entries totaled 21 items and were rated on a 5-point Likert scale, with 1 to 5 indicating strongly disagree ∼ strongly Agree, respectively. The scores were positively related to professional identity. This scale is widely used in the Chinese population. The Cronbach's alpha coefficient for the scale in this study was 0.969.

### 2.3. Data Collection

This involved explaining the purpose and method of questionnaire distribution to the heads of the nursing management in each hospital. After obtaining consent, you can fill out the questionnaire using the Internet, and you will be limited to one response per IP address. The questionnaire included an omission prompt to ensure the completeness of the information. Data in the web backend were exported if they were not updated for one consecutive week. The two researchers jointly verified and excluded invalid questionnaires; 805 valid questionnaires were returned, with a valid recovery rate of 94.15%. The study used a rough estimation of the sample size, and with the addition of approximately 20% meaningless questionnaires, the maximum number was 252, which was sufficient for this study.

### 2.4. Ethical Considerations

The study was approved by the Ethics Committee of Bengbu Medical University, and all participants provided informed consent before the investigation. The ethical approval number was Grant no. 2023369.

### 2.5. Statistical Analysis

The CD-RISC-10 is scored on a 5-point scale. In particular, with a value < 3 as the lower responder. The low responder group was estimated to be 0. Furthermore, a value ≥ 3 was the higher responder group, estimated as 1. AAQ II was scored on a 7-point scale, with ≥4 categorized as a low responder group and estimated as 0. In addition, a value < 4 was categorized as a higher responder group and estimated as 1. After the scores were standardized to dichotomous categories, potential category analysis was conducted using the Mplus 7.4 software. We used a range of test values to explore the most superior classification [24]. Akaike's information criterion (AIC), Bayesian information criterion (BIC), and corrected Bayesian information criterion (aBIC) with lower values indicated a better fit. When the likelihood ratio (LMR) and bootstrap-based likelihood ratio test (BLRT) are significant (*p* < 0.05), the classification of the k-class model is better than that of the *k*-1 class. Regarding the classification accuracy, the closer the entropy value is to 1, the better. More importantly, the final choice of the model must be fully observed for its practical implications [25].

Data were statistically analyzed using the SPSS software (version 26.0). Frequencies and percentages indicate qualitative data, while quantitative data obeying normal distribution are expressed as mean ± standard deviation ($\overset{¯}{x}\pm s$). Comparisons of scores between scales and modalities were performed using analysis of variance (ANOVA), and unordered categorical comparisons were performed using chi-square tests. The Fisher–Freeman–Halton test was used to measure the underfrequency of a particular cell, and the Kruskal–Wallis test was used to measure differences in continuous variables. Finally, multivariate logistic regression was used to analyze the factors influencing nurses' resilience and flexibility categories, which were statistically significant at *p* < 0.05.

## 3. Results

### 3.1. General Demographic Characteristics

A total of 805 nurses participated in this study. The results are summarized in Table 1. More than one-third were 30 years of age or younger (34.2%), primarily female (92.2%), and married (71.4%). 359 nurses (44.6%) had only one child, 412 nurses (51.0%) were mid-level, only 74 nurses (9.2%) were care managers, 205 nurses (25.5%) had been working for one to five years, 609 nurses (75.7%) had a bachelor's degree, 402 nurses (49.9%) were contracted employees, 383 nurses (47.6%) earned between $5001–7000 per month, and 640 nurses (79.5%) worked in a tertiary care hospital as shown in Table 2.

### 3.2. Scale Scores

Resilience (37.96 ± 7.34); psychological flexibility (26.91 ± 10.75); growth mindset (11.35 ± 3.83); and nurses' professional recognition dimensions and total scores were 35.07 ± 6.34, 16.57 ± 3.73, 15.54 ± 4.12, 25.62 ± 6.12, 22.1 ± 4.54, and 114.9 ± 22.32 points.

### 3.3. Latent Classes of Resilience and Psychological Flexibility

Exploratory latent class analyses were based on nurses' resilience and flexibility dichotomous scores. First, 1–5 models were established. The values of AIC, BIC, and aBIC in the model tended to be minor, and the LMR of the five classifications was >0.05. Therefore, they were not considered. For the four categorical models, when both LMR and BLRT were significant, the curves of the four classifications were more complex, somewhat ambiguous, and challenging to interpret in terms of their theoretical and practical significance. By observing the three-category model with an entropy of >0.8 and both LMR and BLRT of <0.05, the categorization is concise, and its functional relevance is more apparent. The results are summarized in Table 1. Ultimately, the best latent class was determined to be three.

### 3.4. Characterization and Naming of Different Models

**Figure 2:**
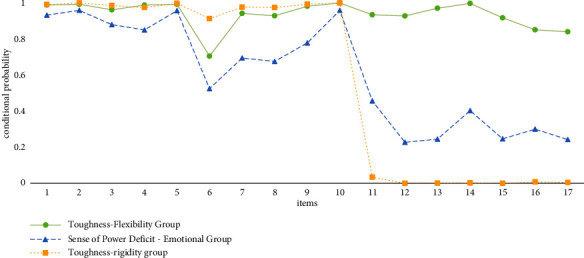
Conditional probability distributions for latent classes of resilience and mental flexibility.

Of the three classification schemes identified, the first group was named the “toughness-flexible group,” which accounted for 32.8% of the total. In this group, the probability of scoring most items for resilience and mental flexibility was above 0.8, indicating that nurses in this group were more resilient and flexible. The second group was categorized as the “power-deficit-emotional group,” which accounted for 23.1%. The perceived sense of strength against adversity was low, and the low-scoring entries in flexibility were emotional concerns and worries that led to stumbling. The third group was categorized as the “toughness-rigid group,” which accounted for 44.1% of the total. This group is characterized by a degree of resilience but generally poor mental flexibility as shown in Figure 2.

### 3.5. Results of One-Way Analysis of Latent Classes of Resilience and Psychological Flexibility

Comparisons of nurses in the different resilience and psychological flexibility categories regarding monthly income, job title, and mode of employment revealed significant differences. The results are summarized in Table 3.

### 3.6. Comparison of Growth Mindset and Professional Identity Scores for Nurses in Three Latent Classes

A comparison of nurses in the three potential categories of resilience and psychological flexibility in terms of growth mindset and total professional identity scores revealed statistically significant differences (*p* < 0.05), as shown in Table 4.

### 3.7. Multiple Logistic Regression Analysis of Factors Influencing Latent Classes of Nurses' Resilience and Psychological Flexibility

Unsorted multicategorical logistic regression analyses were conducted using the potential categories of nurse resilience and psychological flexibility as dependent variables (with the toughness-flexible group as the reference) and variables statistically significant in the univariate analyses as independent variables and covariates. The results indicated that contract employment style was a predictor of the strength-deficit-emotional group; monthly income < RMB5,000, staffing agency, contract employment style, and growth mindset were predictors of the toughness-rigid group; and higher occupational identity and growth mindset together predicted the toughness-flexible group, as shown in Table 5.

## 4. Discussion

### 4.1. Monthly Income Mode of Employment can Affect Nurses' Resilience and Psychological Flexibility

Nurses earning less than RMB 5,000 per month were more likely to be in the rigid toughness group than in the flexible toughness group. In this group, nurses with families and children accounted for more than 2/3, and for their family consumption, a monthly income of less than 5,000 yuan was slightly constrained. An Australian study [26] states that low-income families may be more likely to adopt a child-focused spending plan that allocates less to the adult budget and often requires careful budgeting to meet the family's needs. The dual pressures generated by work and life lead to looking ahead and empirical avoidance when addressing other challenges. Psychological flexibility mediates perceived stress and negative emotions [27]. Based on the family systems' theory, poorer parental psychological flexibility leads to poorer family functioning [28]. For nurses with families and children, the more negative emotions generated, the less family functioning and psychological flexibility.

Contractual hiring practices were an influential factor in the strength-deficit-emotional group compared to the toughness-flexibility group. Contractual employment is one of the most common methods of recruiting nurses. However, this may make nurses feel more apprehensive. In certain cities in China, other modes of employment represent different jobs, hospital benefits, and professional statuses. In contrast, nurses employed on a contractual basis have relatively low educational qualifications, monthly incomes, and retirement benefits. Dr Kate interviewed nurses working temporarily in the UK, who reported feelings of isolation, fewer training opportunities, and difficulties in maintaining permanent jobs [29]. According to the job demand resource model [30], when job resources are limited, demand fulfillment frustration occurs, and personal commitment to work decreases, which may ultimately lead to burnout or separation from service [31].

Personnel agencies and contractual hiring practices affected the toughness-rigidity group. This may have had a different influence on the two outcomes of toughness and mental rigidity in this group. The better flexibility in this group may have resulted from the personnel agency method of employment. A personnel agency is a new type of personnel management in which the Chinese government provides social services for talent. [32]. Compared with other forms of employment, staffing agencies consider the advantages of legal protection, flexible employment, and reasonable distribution of performance pay, among others. Most contractors are highly educated and qualified [33]. This implies favorable future career development and confirms the protective role of the professional dimension in the shield layer of resilient shield theory.

### 4.2. Growth Mindset as an Influence on Resilience and Flexibility of Nurses' Mindset

A growth mindset is a factor influencing the “tough-flexible” group and the “tough-rigid” group. A similar characteristic of both groups was a high level of resilience. After several major public health events, the nursing community underwent significant changes in professionalism and psychological adaptation [34]. Mental health and occupational psychology have received worldwide attention [35]. To prevent and recover emotional resilience from burnout, Jay encourages cultivating a growth mindset that can be used to enhance emotional resilience through the lens of positive psychology [36]. Raquel demonstrated significant correlations and predictions between psychological flexibility and resilience to minimize burnout and trauma among Spanish nurses during major public health events [37]. Growth mindset and mental flexibility, while not practical in prediction, showed statistically significant differences in growth mindset in at least two groups in one-way outcome analyses. In short, the predictive effect of a growth mindset in this study appears to be related to nurses' resilience, and its relationship with psychological flexibility needs to be further demonstrated.

### 4.3. Professional Identity Affects Nurses' Psychological Resilience and Flexibility

Professional identity predicts toughness and flexibility. Zhou et al. concluded that nursing students with a high level of professional identity had positive perceptions and evaluations of the nursing profession, and their capacity for self-directed learning was higher [38]. Research has also demonstrated that people with higher levels of self-directed learning also tend to be more personally resilient [39]. Conversely, Chen further elucidated the critical role of resilience in work-family conflict and career development in his study, explaining why occupational identity is a high resilience predictor [40]. Chong et al. [41] conducted an online survey of 514 Hong Kong citizens whose sequential equation modeling showed that psychological flexibility directly affected prosocial behavior. Occupational identity includes how the wider society recognizes the work an individual does and is a way of demonstrating the individual's relationship with society [42]. Professional identity includes a great deal of prosocial identity.

### 4.4. Limitations

Our study had some limitations. First, our sample comes from only one province in Central China, and economic development and humanistic literacy differ across regions, which may result in a certain degree of bias. Second, there were more female nurses in the sample, which may have prevented comparisons from considering gender differences. Third, the sample size of tertiary care hospitals was large, and differences between different hospital levels may not have been available. Future studies should expand the sample to a national or multinational scale to extrapolate the results.

## 5. Conclusions

This is the first study to simultaneously examine the relationship between nurses' resilience and psychological flexibility. Moreover, this study empirically demonstrates the effects of a growth mindset and professional identity on psychological resilience and flexibility. According to our study, higher career identity predicted the toughness-flexibility group, a better growth mindset predicted high flexibility, and the relationship with psychological flexibility needs further validation. Different modes of employment and income levels are equally important factors affecting the resilience and flexibility of nurses. With the current significant nursing shortage, fair and varied career development for the nursing community is a must for healthcare providers.

### 5.1. Implications for Nursing Management

The percentage of poorly adapted groups in the different models explored in this study was 67.2%, indicating that more resilient nurses may experience poor psychological flexibility. By examining variables related to nurses' career development, we found that improving professional identity and a growth mindset contribute to the psychological resilience and flexibility of the nurse population. This also reflects the fact that good personal growth and professional development are beneficial for nurses' adaptive mindsets. By synthesizing work-related characteristics, nursing administrators and related leaders can make the following attempts: first, enhancing nurses' sense of professional identity and social recognition. Examples include education about the significance of the profession, a sense of respect, and the exploration of multiple channels to build career development paths and display platforms. Second, nurses need to develop a growth mindset, such as teaching practices and learning experiences that support personal development, competency-based medical education, matching vocational assessment programs to nurses' professional needs and purposes, and managers practicing encouraging language and building supportive interactions. Third, employment mechanisms should be reformed, job security implemented, and equal pay for equal work.

## Figures and Tables

**Table 1:** Potential category model fit indices for nurse resilience and psychological flexibility (*n* = 805).

Model	Log (L)	AIC	BIC	aBIC	Entropy	LMR (P)	BLRT (P)	Probability
1	−5454.491	10942.982	11022.726	10968.742	—	—	—	1
2	−3827.287	7724.575	7888.754	7777.609	0.955	<0.001	<0.001	60.4/39.6
3	−3607.693	7321.386	7570.001	7401.696	0.902	0.0191	<0.001	32.8/23.1/44.1
4	−3438.551	7019.102	7352.152	7126.686	0.929	0.0241	<0.001	27.1/8.6/45.4/18.9
5	−3324.538	6827.077	7244.562	6961.936	0.923	0.1165	<0.001	7.8/44.2/3.6/19.2/25.2

**Table 2:** Demographic characteristics of participants.

Projects		Number	%
Age	<30	276	34.3
	<35	236	29.3
	<41	131	16.3
	≥41	162	20.1

Sex	Female	745	92.5
	Male	60	7.5

Education level	Junior college and below	193	24.0
	Undergraduate	609	75.7
	Postgraduate or above	3	0.4

Marital status	Unmarried	213	26.5
	Married	577	71.7
	Others	15	1.9

Number of children	0	261	32.4
	1	359	44.6
	≥2	185	23.0

Monthly income	<5000 RMB	260	32.3
	<7000 RMB	383	47.6
	≥7000 RMB	162	20.1

Working years	1∼5	205	25.5
	6∼10	178	22.1
	11∼15	204	25.3
	16∼20	82	10.2
	≥21	136	16.9

Position	Nurse	731	90.8
	Nursing managers	74	9.2

Professional title	Junior title	364	45.0
	Intermediate title	412	51.0
	Senior title	29	3.6

Employment relationship	Staffing of government-affiliated institutions	161	20.0
	Personnel agent	242	30.1
	Contractual employment	402	49.9

Hospital level	Contractual employment	640	79.5
	Tertiary hospitals and others	165	20.5

**Table 3:** Univariate analysis of 3 latent classes (*n* = 805, cases (percentage, %)).

Projects		Toughness-flexibility group (*n* = 264)	Sense of power-deficit-emotional group (*n* = 186)	Toughness-rigidity group (*n* = 355)	Statistical value	*P* value
Age	<30	98 (37.1)	57 (30.6)	121 (34.1)	3.919^a^	0.141
	<35	60 (22.7)	53 (28.5)	123 (34.6)		
	<41	46 (17.4)	30 (16.1)	55 (15.5)		
	≥41	60 (22.7)	46 (24.7)	56 (15.8)		

Sex	Female	252 (95.5)	172 (92.5)	321 (90.4)	5.560^b^	0.061
	Male	12 (4.5)	14 (7.5)	34 (9.6)		

Education level	Junior college and below	62 (23.5)	46 (24.7)	85 (23.9)	0.075^a^	0.963
	Undergraduate	201 (76.1)	139 (74.7)	269 (75.8)		
	Postgraduate or above	1 (0.4)	1 (0.5)	1 (0.3)		

Marital status	Unmarried	75 (28.4)	52 (28.0)	86 (24.2)	4.464^c^	0.341
	Married	187 (70.3)	129 (69.4)	261 (73.5)		
	Others	2 (0.8)	5 (2.7)	8 (2.3)		

Number of children	0	93 (35.2)	56 (30.1)	112 (31.5)	1.192^a^	0.551
	1	113 (42.8)	91 (48.9)	155 (43.7)		
	≥2	58 (22.0)	39 (21.0)	88 (24.8)		

Monthly income	<5000 RMB	78 (29.5)	55 (29.6)	127 (35.8)	10.158^a^	0.006
	<7000 RMB	119 (45.1)	89 (47.8)	175 (49.3)		
	≥7000 RMB	67 (25.4)	42 (22.6)	53 (14.9)		

Professional title	Junior title	122 (46.3)	81 (43.6)	161 (45.4)	0.578^c^	0.749
	Intermediate title	133 (50.4)	96 (51.6)	183 (51.5)		
	Senior title	9 (3.4)	9 (4.8)	11 (3.1)		

Working years	1–5	78 (29.5)	41 (22.0)	86 (24.2)	4.177^a^	0.124
	6–10	48 (18.2)	35 (18.8)	95 (26.8)		
	11–15	59 (22.3)	56 (30.1)	89 (25.1)		
	16–20	26 (9.8)	13 (7.0)	43 (52.4)		
	≥21	53 (20.1)	41 (22.0)	42 (11.8)		

Position	Nurse	230 (87.1)	169 (90.9)	332 (93.5)	7.430^b^	0.024
	Nursing managers	34 (12.9)	17 (9.1)	23 (6.5)		

Employment relationship	Staffing of government-affiliated institutions	62 (23.5)	40 (21.5)	59 (16.6)	22.425^b^	<0.01
	Personnel agent	94 (35.6)	62 (33.3)	86 (24.2)		
	Contractual employment	108 (40.9)	84 (45.2)	210 (59.2)		

Hospital level	Tertiary hospitals	201 (76.1)	143 (76.9)	296 (83.4)	5.895^b^	0.052
	Others	63 (23.9)	43 (23.1)	59 (16.6)		

^b^Indicates the *χ*^2^ value. ^a^Shows the *H* value. ^c^Shows that the Fisher–Freeman–Halton test was used.

**Table 4:** Comparison of growth mindset and professional identity scores for nurses in 3 latent classes (scores,$\overset{¯}{x}$ ± *s*).

		Growth mindset scale	Nurses' professional identity rating scale					
Groups	Number	Total	Self-efficacy	Sense of consistency	Sense of self-determination	Sense of patience and organizational	Impact sense of meaningfulness	Total
Toughness-flexibility group	264	11.86 ± 4.095	35.25 ± 6.654	16.76 ± 3.878	15.6 ± 4.089	25.51 ± 6.315	22.25 ± 4.62	123.18 ± 17.919
Sense of power-deficit-emotional group	186	9.86 ± 3.175	35.06 ± 5.735	16.45 ± 3.385	15.66 ± 3.721	25.86 ± 5.462	22.35 ± 4.079	107.82 ± 23.479
Toughness-rigidity group	355	11.75 ± 3.741	34.9 ± 6.421	16.47 ± 3.811	15.42 ± 4.341	25.55 ± 6.321	21.84 ± 4.717	112.46 ± 22.823
*F* value		19.147	0.237	0.551	0.264	0.207	1.016	31.929
*P* value		<0.001	0.789	0.576	0.768	0.813	0.362	<0.001

**Table 5:** Multivariate logistic regression analysis of influencing factors of different potential categories.

		*B*	SE	Wald*χ*^2^	*P*value	OR	95% CI	
Toughness-flexibility group	Position (concerning care managers)							
	Nurse	0.004	0.34	0	0.99	1.004	0.516	1.954
	Monthly income (taking ≥7000 as a reference)							
	<5000	−0.16	0.288	0.307	0.579	0.852	0.485	1.499
	<7000	0.048	0.256	0.035	0.851	1.049	0.635	1.733
	Mode of employment (using the staffing of government-affiliated institutions as a reference)							
	Personnel agent	0.358	0.28	1.631	0.202	1.43	0.826	2.477
	Contractual employment	0.532	0.27	3.892	0.049	1.702	1.003	2.887
	Growth mindset	−0.092	0.029	10.186	0.001	0.912	0.861	0.965
	Professional identity	−0.03	0.005	33.542	<0.001	0.971	0.961	0.981

Sense of power-deficit-emotional group	Position (regarding care managers)							
	Nurse	0.3	0.302	0.987	0.32	1.35	0.747	2.442
	Monthly income (taking ≥7000 as a reference)							
	<5000	0.562	0.277	4.095	0.043	1.753	1.018	3.02
	<7000	0.416	0.253	2.712	0.1	1.516	0.924	2.489
	Mode of employment (using the staffing of government-affiliated institutions as a reference)							
	Personnel agent	0.579	0.243	5.695	0.017	1.785	1.109	2.872
	Contractual employment	0.79	0.233	11.472	0.001	2.204	1.395	3.482
	Growth mindset	0.054	0.024	4.969	0.026	1.056	1.007	1.107
	Professional identity	−0.027	0.005	35.493	<0.001	0.973	0.964	0.982

The toughness-flexibility group is taken as a reference category.

## Data Availability

The data that support the findings of this study are available from the corresponding author upon reasonable request.
