# Correction to “Resilience and Flexibility for Clinical Nurses: A Latent Class Analysis”

**DOI:** 10.1155/jonm/9787456

**Published:** 2026-01-10

S. Zhao, Z. Zhang, X. Duan, et al., “Resilience and Flexibility for Clinical Nurses: A Latent Class Analysis,” *Journal of Nursing Management*, 2024, 6171305, https://doi.org/10.1155/2024/6171305.

In the article, there is an error in Table 5, where “Sense of Power Deficit—Emotional Group” should be swapped with “Toughness—Rigidity Group.” The correct Table 5 is shown below:

**Table 5:** Multivariate logistic regression analysis of influencing factors of different potential categories.

		***B***	**SE**	**Wald** **χ** ^2^	**p** **value**	**OR**	**95% CI**	

Sense of power deficit—emotional group	Position (concerning care managers)							
	Nurse	0.004	0.34	0	0.99	1.004	0.516	1.954
	Monthly income (take ≥ 7000 as a reference)							
	< 5000	−0.16	0.288	0.307	0.579	0.852	0.485	1.499
	< 7000	0.048	0.256	0.035	0.851	1.049	0.635	1.733
	Mode of employment (using the staffing of government‐affiliated institutions as a reference)							
	Personnel agent	0.358	0.28	1.631	0.202	1.43	0.826	2.477
	Contractual employment	0.532	0.27	3.892	0.049	1.702	1.003	2.887
	Growth mindset	−0.092	0.029	10.186	0.001	0.912	0.861	0.965
	Professional identity	−0.030	0.005	33.542	< 0.001	0.971	0.961	0.981

Toughness—rigidity group	Position (regarding care managers)							
	Nurse	0.300	0.302	0.987	0.320	1.350	0.747	2.442
	Monthly income (take ≥ 7000 as a reference)							
	< 5000	0.562	0.277	4.095	0.043	1.753	1.018	3.02
	< 7000	0.416	0.253	2.712	0.100	1.516	0.924	2.489
	Mode of employment (using the staffing of government‐affiliated institutions as a reference)							
	Personnel agent	0.579	0.243	5.695	0.017	1.785	1.109	2.872
	Contractual employment	0.790	0.233	11.472	0.001	2.204	1.395	3.482
	Growth mindset	0.054	0.024	4.969	0.026	1.056	1.007	1.107
	Professional identity	−0.027	0.005	35.493	< 0.001	0.973	0.964	0.982

*Note:* Toughness–Flexibility Group as a reference category.

We apologize for this error.

